# Treatment of partial thickness burns of the face with Acticoat7™: A retrospective single center study

**DOI:** 10.1007/s00508-020-01757-z

**Published:** 2020-10-30

**Authors:** Jakob Nedomansky, Alan Oramary, Stefanie Nickl, Gunther Fuchs, Christine Radtke, Werner Haslik, Alexandra Fochtmann-Frana

**Affiliations:** 1 Division of Plastic and Reconstructive Surgery, Department of Surgery, Medical University of Vienna, Währinger Gürtel 18–20, Vienna, Austria; 2 Division of Plastic and Reconstructive Surgery, University Hospital of St. Pölten, St. Pölten, Austria; 3 Department of Plastic, Reconstructive, Hand, and Burn Surgery, StKM—Klinikum Bogenhausen, Academic Teaching Hospital Technical University Munich, Munich, Germany; 4 Department for Plastic, Aesthetic and Reconstructive Surgery, State Hospital Wiener Neustadt, Wiener Neustadt, Austria

**Keywords:** Burn injury, Facial burns, Silver dressing, Thermal injury, Intensive care unit

## Abstract

**Background:**

The face is affected in more than 50% of patients with extensive burn trauma. Effective treatment is of importance to avoid hypertrophic scarring, functional impairment and social stigmatization.

**Material and methods:**

All patients treated with Acticoat7™ due to superficial and deep partial thickness burns of the face between 2008 and 2017 at the intensive care unit (ICU) for burn trauma at the Department for Plastic and Reconstructive Surgery of the Medical University of Vienna were retrospectively analyzed. Patients were evaluated for the number of required dressing changes until complete re-epithelialization, bacterial colonization, potential complications and the need for primary and secondary surgery.

**Results:**

A total of 100 patients were analyzed. It took a median dressing change rate of 1 (range 0–5) in the superficial partial thickness and 3 (range 1–11) in the deep partial thickness group. Conservative treatment of deep partial thickness wounds was possible in 79% and 17% of these patients required secondary scar revision. Although bacterial colonization of the wounds frequently occurred, wound infections were rarely observed.

**Conclusion:**

Acticoat7™ is a valuable dressing for treating superficial and deep partial thickness burn wounds of the face in an intensive care unit setting. It enables extended time intervals between dressing changes without an increased risk for complications.

## Introduction

Facial burns occur in more than 50% of patients with extensive burn trauma. The face represents a fundamental part of the personal identity and is of great importance for social interaction. Therefore, treatment of facial burns represents a versatile challenge [1].

Due to the high potential for spontaneous healing of facial burns, early surgical excision of the burn eschar followed by skin grafting is often not indicated as the first-line treatment option [2]. Effective conservative treatment of partial thickness burn wounds of the face is important to avoid delayed wound healing and decrease the risk for hypertrophic scar formation [3]. Hypertrophic scarring in the face often leads to functional limitations (e.g. incomplete closing of the eyelids and eating problems) and social stigmatization [4]. The three-dimensional contour of the face and its steady movements impose high demands on burn dressings used in this area [5]. In the past topical ointments (e.g. Flamazine™ [Alliance Pharmaceuticals GmbH, Düsseldorf, Germany]) were used at many burn centers but they were associated with extended healing times [6]. Nowadays, there is a wide range of alternatives available for partial thickness burns (e.g. antimicrobial silver dressings, hydrocolloid dressings, bioengineered skin substitutes, polyurethane film dressings, hydrogel dressings and silicon-coated nylon dressings) and compared to topical ointments, most of them require fewer dressing changes before complete re-epithelialization takes place [7].

Acticoat7™ (Smith & Nephew GmbH, Hamburg, Germany) is a nanocrystalline silver dressing, which consists of a rayon/polyester core and a silver-coated high-density polyethylene mesh. Acticoat7™ provides constant release of silver for up to 7 days and therefore can be left on the wounds for up to 7 days [8, 9]. The main goal of this study was to retrospectively evaluate outcomes after conservative treatment of superficial and also deep partial thickness burns of the face with Acticoat7™.

## Patients, material and methods

### Study design and population

This retrospective study analyzed data from all patients with superficial and deep partial thickness facial burns treated at the 6‑bed intensive care unit (ICU) of the Vienna General Hospital between 2008 and 2017. All patients included were treated with Acticoat7™, a wound dressing containing nanocrystalline silver. The data were obtained from scanned patient charts. Approval from the local ethics committee (No. 1183/2017) was obtained. Patients transferred to the burn unit after initial treatment at external hospitals, patients who died during the course of treatment and patients suffering from third degree facial burns were excluded. The primary endpoint was determined as surgery required or not. A secondary endpoint was determined as need for secondary scar revision.

### Initial wound management

**Fig. 1:**
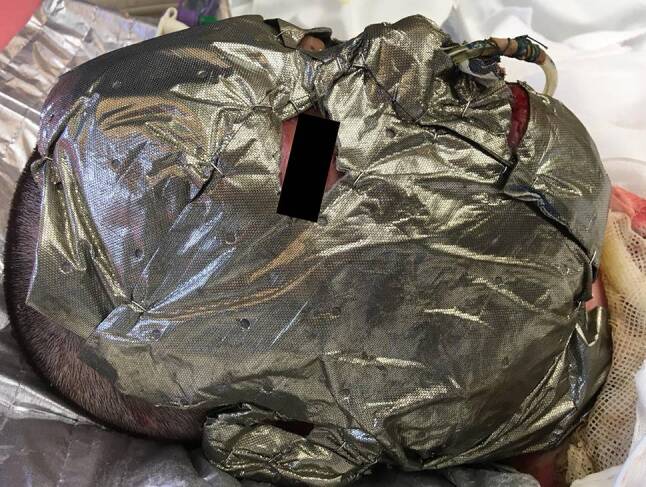
Applied Acticoat7™ mask fixed with skin staples

All patients admitted to the ICU for burn trauma were evaluated for the extent and depth of the burn wounds. In cases of involvement of the face hair of the scalp and beard were shaved. Blisters were removed and the wounds were treated with an antiseptic agent (Lavasorb®, Fresenius Kabi AG, Bad Homburg, Germany) and digital photographic documentation was performed. A senior physician determined the burn depth by bedside examination on the day of admission. Finally, Acticoat7™ was applied and fixed with small skin staples (see Fig. 1).

### Further wound management

Acticoat7™ was moisturized in the course of the treatment with sterile distilled water twice a day. Dressing changes were performed after 7 days. When a dressing change took place a mechanical cleaning of the wounds was performed. Necrotic tissue was removed by means of moist compresses and the wounds were cleaned, shaved, and evaluated for the necessity of further Acticoat7™ treatment.

in the case of re-epithelialization, Acticoat7 treatment was terminated and moisturizing ointments were recommended. In our institution all burn patients had follow-up appointments on a regular basis (6 weeks, 3 months, 6 months, 1 year and 2 years after discharge and after that every year). Close outpatient follow-up visits were required to regularly check on the process of wound healing, compression clothes, and scar formation. When required secondary scar revision was conducted.

### Obtained data

We analyzed demographic data (age, sex and trauma mechanism), burn depth, affected total body surface area (TBSA), days until healing, bacterial colonization, wound infections, concomitant trauma and need for secondary surgical scar correction.

### Statistical analysis

Demographic data, such as sex, trauma mechanism, bacterial colonization, depth of the burn wound and complications are presented in total numbers and percentages. Metric values, such as age, days until completed healing, number of dressing changes, percentage of burned body surface area, are presented as mean ± standard deviation and median values (minimum-maximum). The normality of distribution was determined using the Shapiro-Wilk test. All statistical analysis was performed with SPSS 15.0.1 for Windows (IBM, Armonk, NY, USA).

## Results

### Patient characteristics

A total of 100 patients who underwent treatment of superficial and deep partial thickness burns of the face with Acticoat7™ were included of which 64 were women and 36 men with an average age of 46 ± 18 years (Table 1).

**Table 1:** Patient characteristics

Characteristic	*N* = 100
*Age (years)*	
Mean ± SD	46 ± 18
*Sex*	
Male	36
Female	64
*TBSA (%)*	
Median (minimum-maximum)	25 (2–80)
*Burn depth*	
Superficial partial thickness	62%
Deep partial thickness	38%
*Trauma mechanism*	
Combustion	84%
Scalding	11%
Electrical burn	4%
Chemical burn	1%

*TBSA* total body surface area, *SD* standard deviation

### Burn depth, extent and additional trauma

Of the patients 62 (62%) had superficial and 38 (38%) had deep partial thickness burns. Median affected TBSA was 25% (range 2–80%). Additional trauma to the eyes and the respiratory system occurred in 56% and 27%, respectively.

### Healing time superficial partial thickness (IIa) wounds (*n* = 62)

Median healing time was 13 days (range 6–32 days) with a median dressing change rate of 1 (range 0–5). None of the superficial partial thickness wounds required primary or secondary surgery. The number of dressing changes and healing times were not documented in 8 patients (13%, Table 2).

**Table 2:** Outcomes of conservative treatment in different burn depths

*Outcome*	*Superficial partial thickness* *n* *=* *62*	*Deep partial thickness* *n* *=* *38*
*Healing time (days)*		
Median (min-max)	14 (6–42)	24 (9–88)
*Dressing changes*		
Median (min–max)	1 (0–5)	3 (1–11)
*Missing data*	8 patients	5 patients

### Healing time deep partial thickness (IIb) wounds (*n* = 38)

Of the patients with deep partial thickness wounds 30/38 (79%) were treated solely conservatively with a median healing time of 24 days (range 9–88 days) and a median of 3 (range 1–11) dressing changes (Table 2). Of the patients 8/38 (21%) were initially treated with Acticoat7™ and required surgery during the further course of treatment. The surgical treatment (debridement and skin or keratinocyte grafting) was necessary due to wound infections or prolonged healing time. Median total healing time for these patients was 34 days (range 15–61 days, conservative treatment and postoperative period).

### Secondary scar revision

Of the 30 patients with deep partial thickness burns, who were treated conservatively 5 (17%) required secondary scar revision. Functional areas impaired by hypertrophic scarring were the eyelids and the mouth (*n* = 1 eyelid, *n* = 1 eyelid and mouth and *n* = 3 mouth). None of the patients with superficial partial thickness burns developed hypertrophic scarring leading to functional impairment.

### Bacterial colonization

**Fig. 2:**
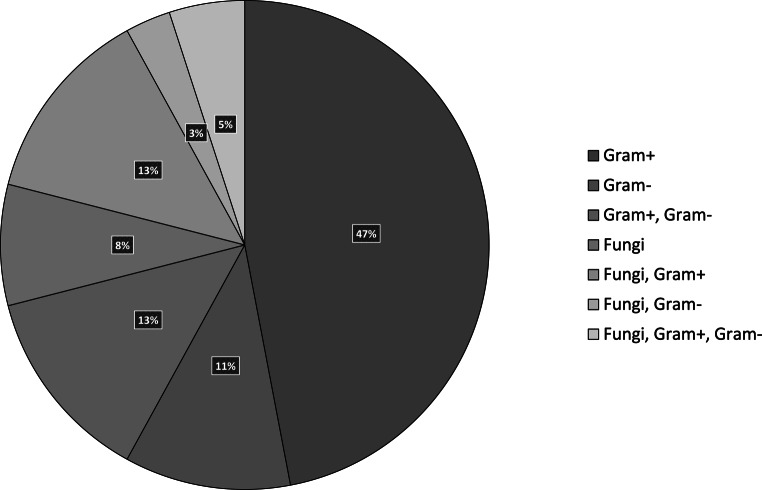
Types of microorganisms detected in wound swabs taken from the facial area

Wound swabs were taken in 48% of the patients and 79% of these swabs were positive. The majority (63%) of microbiological analysis of wound swabs taken from the facial area showed colonization with more than one microbial agent (Fig. 2). Multidrug-resistant (MDR) bacteria were detected in wound swabs of two patients. One patient showed growth of methicillin-resistant *Staphylococcus aureus* (MRSA) and another patient showed growth of carbapenem-resistant *Pseudomonas aeruginosa* 3MRGN (multidrug-resistant gram-negative pathogens with resistance to three antibiotic groups [defined by the German Commission for Hospital Hygiene and Infection Prevention]). The types of microorganisms detected in wound swabs taken from the facial area are shown in the pie chart in Fig. 2.

## Case reports

### Case report 1

**Fig. 3:**
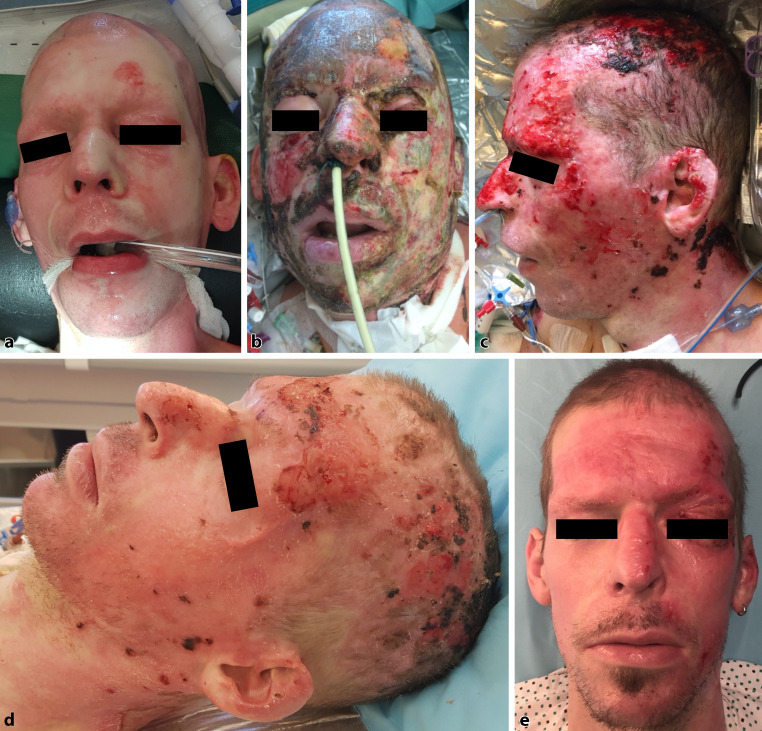
Patient presented in case report 1. **a** Facial burn injury on day of admittance, **b** result after 7 days of treatment, **c** result after 35 days of treatment, **d** result after 49 days of treatment and **e** result after complete re-epithelization and scar revision with a full thickness skin graft due to incomplete eye closure of the left eye

A 41-year-old male patient was admitted to the burn unit with deep partial thickness and full thickness burns covering 25% of the TBSA including the face (Fig. 3a). The patient, who had a history of chronic nicotine and intravenous drug abuse, suffered these injuries during a fire in his apartment. After initial wound cleaning, Acticoat7™ treatment was started. Dressing changes were performed every 7 days and swabs were taken during each dressing change (Fig. 3b, c). The microbiological analysis of the wound swabs showed growth of MRSA, *Streptococcus parasanguinis, Klebsiella pneumoniae, Enterococcus faecalis, Candida albicans* and *Escherichia coli*. Treatment required a total of 7 dressing changes with at total healing time of 54 days. The prolonged healing time lead to hypertrophic scarring in the left periorbital region resulting in incomplete eyelid closure. A full-thickness skin graft from the clavicular region was used to release the scar contracture (Fig. 3e).

### Case report 2

**Fig. 4:**
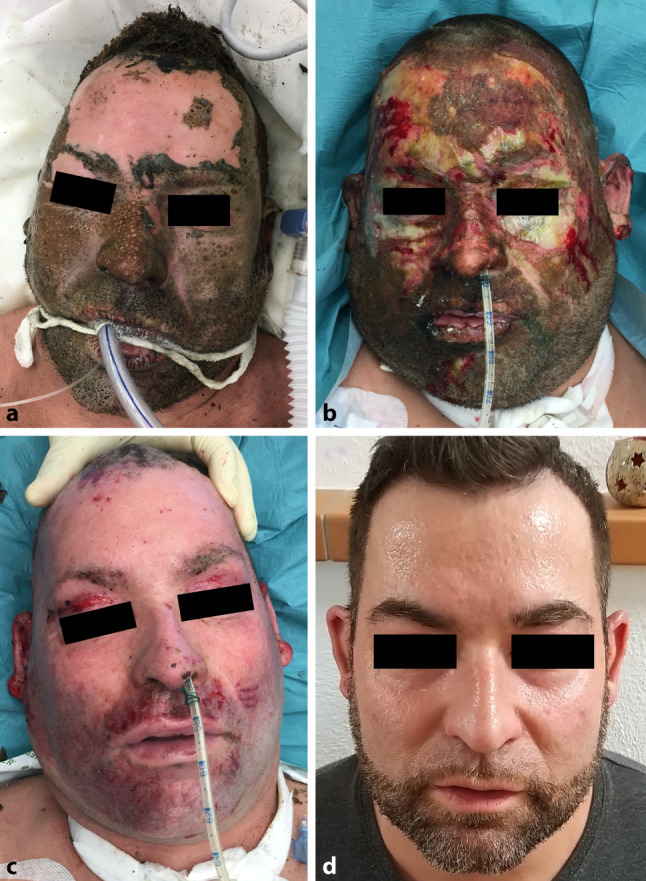
Patient presented in case report 2. **a** Facial burn injury on day of admittance, **b** result after 7 days of treatment, **c** result after 14 days of treatment and **d** result 18 months after the trauma

A 38-year-old male patient was admitted to the burn unit with superficial and deep partial thickness burns covering 20% of the TBSA (Fig. 4a). He suffered the injuries doing construction work on a windmill when a fire broke out. Both upper extremities and the face were affected and Acticoat7™ treatment was started after initial wound cleaning. The facial wounds healed within 14 days requiring only 1 dressing change (Fig. 4b, c and d).

### Discussion

Nanocrystalline silver-impregnated dressings such as Acticoat7™, provide a steady release of long lasting antimicrobial silver ions to the wound bed while overcoming certain significant drawbacks of formerly used silver dressings [10, 11]. The possibility to create a full face mask which can be left on the wound bed for up to 7 days (Fig. 1) made Acticoat7™ the preferred dressing for facial burns at our burn ICU, given that patients required sedation and mechanical ventilation on admittance.

Whereas the clinical efficacy of Acticoat7™ has been the focus of interest in adults with superficial partial thickness burns [12] as well as in pediatric populations with different burn depths [13–15] and also in several previous case reports [16, 17], our aim was to evaluate its value in the conservative treatment of superficial and especially deep partial thickness wounds of the face.

A limitation of the present study is the monocentric, retrospective study design without a control group; however, in our institution, primary surgery is only conducted on full thickness facial burns. The combination of the outstanding tendency for spontaneous healing after facial burn trauma contrasting the relatively frequent esthetically unpleasing results after skin transplantations in the face, led to the conservative treatment plan for deep partial thickness burns of the face at our intensive care unit. The wait and see approach in the acute phase of deep partial thickness burns was effective in the vast majority of patients (79%) with median healing times of less than 4 weeks only requiring 1 dressing change per week after initial application. In several patients delayed primary surgery had to be performed. Indications for delayed primary surgery were wound infections and absence of complete re-epithelialization. Reducing frequencies of primary surgeries of the face decreased patient morbidity and treatment costs in the acute phase. In most patients with deep partial thickness wounds who were treated conservatively, spontaneous healing did not lead to functionally impairing hypertrophic scarring. Only 17% of these patients required secondary scar corrections due to disturbing scarring in the eyes, mouth and chin area. In summary, conservative treatment of deep partial thickness burns of the face without secondary scar revision was possible in two thirds of the cases, whereas one third of patients with deep partial thickness burns of the face required either delayed primary or secondary surgical intervention; however, due to the study design it is impossible to know if the good outcome and the relative low number of surgical interventions was only achieved by the Acticoat7^TM^ treatment. In the present data there is no evidence that only Acticoat7^TM^ has that effect and other dressing materials have not got a similar effect; however, we strongly believe that the treatment of deep partial thickness facial burns should be conservative. Even the most skilled burn surgeon cannot guarantee to preserve every vital dermal cell. Acticoat7^TM^ has a favorable texture and can be modelled as a full face mask. In addition to that it is cost-efficient and does not require a secondary dressing. Furthermore, there is no need for daily dressing changes; however, Acticoat7^TM^ still allows wound inspections, swabs, and debridement with a maximum time frame of 7 days, whereas other dressing material such as Suprathel® (Polymedics Innovations GmbH, Denkendorf, Germany) always requires previous surgical necrosectomy and is left on the wound bed until it peels off by itself. We believe that Acticoat7^TM^ is the most suitable silver dressing for the conservative treatment of facial burns. Especially in deep partial thickness burn wounds a dressing with high potency against microbial agents is necessary and Suprathel® does not provide this antimicrobial potency [18].

Healing times in the superficial partial thickness group were comparable to the results of Verbelen et al. in their study comparing Acticoat7™ with Aquacel®Ag (ConvaTec GmbH, München, Germany), with a mean healing time of 16 days for superficial partial thickness wounds in the Acticoat7™ group [12]. In our patient collective superficial partial thickness wounds healed without short-term or long-term complications, such as hypertrophic scarring.

Wound infections rarely occurred (none in the superficial partial thickness group and five in the deep partial thickness group) although analysis of wound swabs showed a high percentage of bacterial colonization (68% superficial partial thickness, 88% in deep partial thickness). The difference in infection rates between the two groups could be due to a better antibacterial efficacy of the silver ions in the well-perfused superficial partial thickness wound bed. Microbial overgrowth leading to infections only occurred in the deep partial thickness group. These wounds might be more susceptible to infection due to restricted blood perfusion and subsequent immunosuppression. In the case of clinically proven wound infection in combination with a positive wound swab result, antibiotic treatment was initiated or adapted. To ensure early detection of wound infections in burn patients with deep partial thickness facial burns, dressing changes should probably be performed more frequently than every 7 days; however, a more frequent dressing change could potentially compromise the sensible process of re-epithelialization and might therefore slow down the healing process. Therefore, based on the present study findings a general recommendation regarding time interval between the dressing changes cannot be made.

The thought of using skin staples to secure a dressing in the face might be disturbing at first because of the fear of additional scarring. At follow-up examinations of our patient’s visible sequela due to the skin staples were never seen. The face shows a distinctive tendency for swelling within the first hours and days after the burn trauma. Therefore, the first Acticoat7™ mask ought to be applied more loosely and sometimes removal of skin staples is necessary in the occurrence of swelling.

In the following treatment course Acticoat7™ masks should be moisturized twice daily and changed every 7 days. These long intervals between dressing changes avoid unnecessary disruption of the healing process.

Burn depths were always determined clinically by an experienced burn surgeon. Exact healing times could not be determined since the dressing changes were only performed every 7 days. Therefore, the presented numbers are supposedly higher than the actual healing times; however, we believe that the number of necessary dressing changes is a valuable surrogate parameter to evaluate the efficacy of the dressing.

## Conclusion

The fact that the last primary surgery of a deep partial thickness wound of the face at our ICU was performed in 2012, supports our hypothesis that Acticoat7™ allows effective conservative treatment of these kinds of wounds with low primary complication rates and a low percentage of secondary scar revisions.
